# Chromosome-scale genome of European anchovy (*Engraulis encrasicolus* L., 1758) and comparative genomics uncover clupeid-specific immune gene expansions

**DOI:** 10.1093/dnares/dsag009

**Published:** 2026-07-15

**Authors:** Tuana Öğretici, Selahattin Barış Çay, Onur Obut, Mehmet Ali Balcı, Yusuf Ulaş Çınar, Pınar Akbaba, Yasemin Aydın, Banu Orta Yılmaz, Nazlı Kasapoğlu, Şirin Firidin, Fatih Dikmen, Cem Dalyan, Taner Yıldız, Esma Gamze Aksel, Gökmen Zararsız, İlhan Aydın, Yakup Bakır, Melike Erkan, Vahap Eldem

**Affiliations:** Institute of Graduate Studies in Sciences, Istanbul University, Istanbul 34134, Türkiye; Institute of Graduate Studies in Sciences, Istanbul University, Istanbul 34134, Türkiye; Institute of Graduate Studies in Sciences, Istanbul University, Istanbul 34134, Türkiye; Institute of Graduate Studies in Sciences, Istanbul University, Istanbul 34134, Türkiye; Institute of Graduate Studies in Sciences, Istanbul University, Istanbul 34134, Türkiye; Institute of Graduate Studies in Sciences, Istanbul University, Istanbul 34134, Türkiye; Department of Biology, Istanbul University, İstanbul 34134, Türkiye; Department of Biology, Istanbul University, İstanbul 34134, Türkiye; Central Fisheries Research Institute, Trabzon 61290, Türkiye; Central Fisheries Research Institute, Trabzon 61290, Türkiye; Department of Biology, Istanbul University, İstanbul 34134, Türkiye; Department of Biology, Istanbul University, İstanbul 34134, Türkiye; Department of Fisheries Technology and Management, Faculty of Aquatic Sciences, Istanbul University, Istanbul 34134, Türkiye; Department of Genetics, Faculty of Veterinary Medicine, Erciyes University, Kayseri 38280, Türkiye; Department of Biostatistics, Erciyes University, Kayseri 38039, Türkiye; Drug Application and Research Center (ERFARMA), Erciyes University, Kayseri 38039, Türkiye; General Directorate of Fisheries and Aquaculture, Ankara 06800, Türkiye; Department of Medical Laboratory Techniques, Vocational School of Health Services, Atlas University, Istanbul 34403, Türkiye; Institute of Graduate Studies in Sciences, Istanbul University, Istanbul 34134, Türkiye; Department of Biology, Istanbul University, İstanbul 34134, Türkiye

**Keywords:** European anchovy, chromosome-scale genome, comparative genomics, Gene family dynamics, positive selection analysis

## Abstract

The European anchovy is a keystone forage species in the Mediterranean and Northeast Atlantic, yet a chromosome-scale reference genome has been lacking. Here, we present a 1.4 Gb assembly anchored to 24 chromosomes (scaffold N50 = 56.4 Mb; BUSCO 96.6%) with 27,663 annotated protein-coding genes and 44.6% repetitive content dominated by DNA transposons, which likely account for the near doubling of genome size relative to Atlantic herring. Comparative analyses across 11,755 orthogroups revealed a net contractive trend in Clupeidae, superimposed on conspicuous expansions of antiviral and innate-immune gene families (TRIM16, GTPase IMAP member 8, IFI44, Toll-like receptors), with GO enrichment exceeding 400-fold for antiviral regulatory processes. Branch-site analyses identified 216 positively selected genes in *E. encrasicolus*, 81 of which map to innate signaling, epigenetic regulation, ubiquitin-mediated immunity, and selective autophagy. Multitissue RNA-Seq further demonstrated that expanded, positively selected, and epithelial barrier-associated genes, including 11 claudin paralogues, zonula-occludens scaffolds, and tricellular junction components, are coordinately enriched in the gill. Together, these findings highlight a gill-enriched immune landscape potentially shaped by filter-feeding ecology, providing a genomic framework for clupeid immunogenomics and aquaculture applications.

## 1. Introduction

With more than 430 species, the order Clupeiformes undoubtedly includes the world's most prominent forage fishes, such as anchovies (Engraulidae), scads (Carangidae), and pilchards (Clupeidae), serving as a crucial trophic link among primary producers, zooplanktons, and large-bodied key predators such as piscivorous fishes, seabirds, and marine mammals.^1–3^ These typically abundant small or medium-sized pelagic species are major components of forage fishes in coastal ecosystems and are considered pivotal in structuring marine ecosystems.^4–7^ Clupeiform fishes are ecologically diverse and globally distributed throughout almost all aquatic environments in coastal and open marine environments, estuaries, freshwater, rivers, and lakes.^8,9^ In addition to ecological significance, clupeiforms constitute a considerable portion of the total world fisheries, contributing to global food supply and providing a rich source of animal protein due to exceptionally high nutrient content.^10,11^ Anchovies (*Engraulis* sp.), as small pelagic fishes belonging to the family Engraulidae, are abundant and ubiquitous in the epipelagic zone of the world’s oceans and are a key component of the marine ecosystem due to their high biomass productivity.^12,13^

Filter feeding, a remarkable adaptation observed in Clupeid fishes such as herrings, sardines, and shads, offers a suite of advantages that have contributed to the success and ecological importance of these species.^14^ This efficient feeding strategy allows Clupeids to exploit abundant planktonic food sources with minimal energy expenditure, supporting large populations that play crucial roles in marine ecosystems. By continuously filtering small organisms from the water column, these fish can occupy a distinct ecological niche, reducing competition with other species while maintaining a diverse and steady diet. The adaptability of their gill raker structures enables Clupeids to selectively target specific sizes of plankton and thrive in various aquatic environments, from coastal waters to the open ocean. Understanding the mechanisms and benefits of filter feeding in Clupeid fishes not only sheds light on their evolutionary success but also underscores their significance in marine food webs and fisheries worldwide.^15–17^ While filter feeding offers numerous evolutionary advantages, it also presents challenges for species in the Clupeid fishes. The nonselective nature of filter feeding exposes these fish to a high influx of particulate matter and microorganisms, potentially increasing the risk of pathogen exposure and environmental stress within their gill tissues.^18^ Consequently, the branchial apparatus must possess the capacity to effectively remove harmful particles and pathogens while simultaneously eliciting a robust immunological response to potential threats.^19–21^ This innate defence involves immune cells such as macrophages, neutrophils, and dendritic cells, which initiate phagocytosis and inflammatory responses. The mucosal epithelium on all exposed body surfaces-including skin, respiratory tracts, and the digestive system-serves as a primary defence against external threats, mechanical friction, and dehydration while facilitating the exchange of oxygen and nutrients.^22,23^ Additionally, mucosa-associated lymphoid tissue (MALT) supports adaptive immunity by producing immunoglobulins such as IgM and IgT, crucial for long-term defence against pathogens.^24,25^ Moreover, gill tissues produce reactive oxygen species (ROS) as part of the pathogen neutralization process, while antioxidant enzymes regulate ROS levels to prevent oxidative damage to tissues.^26^

In the context of evolutionary and comparative immunology, Clupeiform fishes (anchovies, herrings, and sardines) present exceptional model organisms for investigating immune responses at the genomic level. Their ecological significance as keystone species in marine food webs and their unique filter-feeding strategy and schooling behavior create a compelling framework for studying immunological adaptations. To bridge the gap between these ecological observations and their molecular underpinnings, high-quality genomic resources are essential. In this study, we generated a chromosome-scale genome assembly of the *E. encrasicolus*, supplemented by detailed transcriptomic annotation to ensure structural and functional accuracy. Leveraging this robust genomic framework, we performed comparative analyses across teleost species to investigate gene family evolution and identify signatures of positive selection, specifically targeting genes involved in immune response and environmental adaptation. Furthermore, we characterized tissue-specific gene expression profiles to validate the functional relevance of these key genes in maintaining homeostasis within the branchial and mucosal systems. To the best of our knowledge, this study represents one of the first comprehensive efforts to elucidate the genomic and evolutionary underpinnings of these adaptations in Clupeiform fishes.

## 2. Materials and methods

### 2.1. Sample collection and DNA extraction

Samples of European anchovies from the East Black Sea region were provided by the Central Fisheries Research Institute of Turkey (Yomra, Trabzon). The samples stored in dry ice were immediately transported to the Molecular Biodiversity Laboratory of Istanbul University. The anchovy samples were acquired from research fishing vessels engaged in monitoring anchovies; therefore, ethical aspects are not applicable. To decrease the heterozygosity level, only one female *E. encrasicolus* individual was used for genomic DNA extraction. High molecular weight (HMW) genomic DNA was extracted from dorsal muscle tissue using the NucleoBond HMW DNA kit (Macherey-Nagel, Germany). The gDNA purity and concentration were determined by NanoDrop 2000c spectrophotometer (Thermo Scientific, USA) and fluorometer Qubit v4 (Thermo Scientific, USA). The integrity of gDNA was checked with agarose gel electrophoresis.

### 2.2. Genome size estimation with genome survey

To estimate genome characteristics including haploid genome length, heterozygosity, and repeat contents, two paired-end read libraries with 180- and 400-bp insert sizes were constructed and sequenced on an Illumina HiSeq2500 platform following the standard manufacturer’s protocol. To get high-quality clean reads, the reads from the Illumina sequencing were filtered by fastp v0.23.2^27^ using the following strategy: (i) discarding sequencing adaptors and low-quality terminal bases (<Q20), (ii) reads with 10 or more unknown bases (Ns) were discarded, (iii) the removal of reads shorter than 30 bp. For genome survey analysis, *k*-mer frequencies (*k* = 19, 21, 23, 25, 27, 29, 31) from these high-quality short reads were counted with Jellyfish v2.3.1,^28^ and the histogram of read *k*-mer abundance was inputted to GenomeScope (http://genomescope.org/genomescope2.0/).^29^

### 2.3. PacBio, stLFR and Hi-C library construction and genome sequencing

Approximately 10 µg gDNA was used to construct the library for PacBio SMRT Sequencing. Firstly, gDNA sample was sheared to an average size of 20 kb using a g-TUBE device, and then sheared gDNA was purified and end-repaired using polishing enzymes. A blunt-end ligation reaction followed by exonuclease treatment was applied to generate a SMRTbell template according to the PacBio 20-kb template preparation protocol. The size selection and quality analysis of the SMRTbell library was carried out by a BluePippin system (Sage Science, USA) to enrich large (>10 kbp) fragments. The constructed libraries were sequenced using the PacBio Sequel II System with CLR mode. The HMW gDNA was also used to construct an stLFR (single-tube long fragment reads) library using MGIEasy stLFR library preparation kit, and then the stLFR library was sequenced with the 2 × 100 bp paired-end module on the DNBSEQ G400 platform (MGI, China). By adding the same barcode sequences to sub-fragments of the DNA (called “co-barcoding”), the stLFR v1.3 technology allows the generation of long-reads for contig scaffolding and haplotyping.^30^ To further assemble, including ordering and orienting scaffolds into chromosome groups, Hi–C library preparation was carried out in several steps, including cross-linking the HMW gDNA, MboI restriction enzyme digestion, end repair, DNA cyclization, and DNA purification. The Hi–C library was sequenced with the 2 × 150 bp paired-end module on the DNBSEQ G400 platform (MGI, China). All sequencing was performed by Beijing Genome Institute (Shenzhen, China). Quality assessment of the raw MGI reads was performed using MultiQC v1.13.^31^ The short reads were first filtered for quality (QC > 20) and length (>30 bp), and adaptor sequences were removed with fastp v0.23.2^27^ using default settings.

### 2.4. *De novo* genome assembly, quality assessment and synteny analysis

For de novo assembly of *E. encrasicolus*, the PacBio sequence data was corrected with “canu correct” (v2.2 with useGrid = true; minThreads=28; genomeSize=950 m; minOverlapLength=700; minReadLength = 1,000).^32^ Next, we assembled Canu-corrected reads using wtdbg2 v2.5.1^33^ and primary contigs of each assembly were polished with NextPolish v1.4.1^34^ with 3 rounds of alignment with high-quality Illumina reads. To enhance the initial genome assembly, SLR-superscaffolder v.1.0^35^ was employed to bridge gaps in the PacBio-derived draft assembly using high-quality paired-end stLFRs, with a read length of 100 bp. This process leveraged both co-barcoding and paired-end read information to improve the contiguity of the genome. To enhance the quality of the assembly, gap filling was performed using TGS-GapCloser v1.1^36^ with PacBio long-read sequencing data. Subsequently, to remove haplotypic duplications, the assembly underwent 2 rounds of processing with purge_dups v1.2.5^37^ under default parameters. Finally, chromosome-level assemblies were generated utilizing Hi–C data. Hi–C reads were aligned to the assembled draft genome using Juicer v1.6,^38^ followed by contig mapping to chromosome-level scaffolds using 3D-DNA v.180922^39^ with default parameters. To evaluate the completeness and quality of the reference genome, we employed the Benchmarking Universal Single-Copy Orthologs (BUSCO) v5.1.2 (OrthoDB v10) on the gVolante web server v2.0.0,^40,41^ using a highly conserved orthologous protein set specific to Actinopterygii. Additionally, basic length-based genome metrics, such as N50 and L50, were calculated using the same web tool. The genome assembly was generated using shinyCircos-V2.0.^42^ To investigate syntenic relationships across chromosomes in Clupeids, chromosome-scale scaffolds were aligned using Minimap2 v2.28.^43^ The resulting alignments were visualized with NGenomeSyn v2.029,^44^ revealing high collinearity across the majority of chromosomal regions.

### 2.5. Genome annotation

Repetitive sequences and transposable elements were first annotated to facilitate gene prediction in the assembled *E. encrasicolus* genomes. Both de novo and homology-based pipelines were employed for repeat annotation using RepeatModeler2 v2.0.5^45^ and RepeatMasker v4.1.4.^46^ For de novo annotation, a repeat library was generated using RepeatModeler2 v2.0.5,^45^ which integrates several tools, including RepeatScout v1.0.6,^47^ RECON v1.08,^48^ TRF v4.09.1,^49^ and LTR_FINDER v1.0.7^50^ to identify long terminal repeat (LTR) elements with default parameters. We combined the de novo database with the homolog based repeat database for Teleost in RepBase release26.05^51^ and Dfam release 3.6^52^ database to build the custom library. In the final step, we used RepeatMasker v4.1.4^46^ against our custom library with the “-s” option for a much more sensitive search to mask repeat regions using *rmblast.pl* v2.13.0 as a search engine. Following repeat masking, protein-coding and noncoding gene annotations were comprehensively predicted using the NCBI Eukaryotic Genomic Annotation Pipeline v10.0.^53^ The assembled *E. encrasicolus* genome was submitted to the NCBI RefSeq database^54^ and assigned the accession number GCF_034702125.1. This automated pipeline identified both coding sequences and various noncoding RNA categories, including long noncoding RNAs (lncRNAs), transfer RNAs (tRNAs), ribosomal RNAs (rRNAs), and pseudogenes. The resulting official RefSeq annotation models were subsequently utilized for all downstream comparative genomic analyses.

### 2.6. Gene family analysis and positively selection (PSGs) analysis

To elucidate the dynamics of genome evolution, a comprehensive analysis of gene family expansion and contraction was conducted utilizing proteome and annotation data from 11 species, encompassing 7 teleost fish taxa (*Lepisosteus oculatus*, GCF_000242695.1; *Danio rerio*, GCF_000002035.6; *Gadus morhua*, GCF_902167405.1; *Takifugu rubripes*, GCF_901000725.2; *Scomber scombrus*, GCF_963691925.1; *Solea solea*, GCF_958295425.1; *Sparus aurata*, GCF_900880675.1) and 4 species within the Clupeiformes order (*E. encrasicolus*, GCF_034702125.1; *A. alosa*, GCF_017589495.1; *C. harengus*, GCF_900700415.2; *S. pilchardus*, GCF_963854185.1). The longest transcript for each gene was selected using AGAT v1.5.1.^55^ Gene family expansion and contraction analyses were conducted utilizing Orthofinder v2.5.5,^56^ with sequence similarities determined through comprehensive all-versus-all BLAST searches, resulting in the generation of Orthogroups data. Phylogenetic trees were constructed using single-copy orthologs identified by Orthofinder v2.5.5^56^ and subsequently visualized using IQ-TREE2 v2.3.6.^57^ An ultrametric tree was then generated employing the Ape v5.8 package in R.^58^ Gene gain and loss events across lineages were modeled using a stochastic birth-death process implemented in CAFE5 v5.1.0.^59^ This analysis incorporated the ultrametric species tree and gene family count data derived from Orthofinder v2.5.5.^56^ We performed positive selection analyses following the guidelines outlined by Álvarez-Carretero et al. (2023).^60^ Proteins derived from single-copy orthogroups, which were identified prior to gene expansion/contraction analysis using Orthofinder v2.5.5,^56^ along with their corresponding coding sequences (CDS) from each species, were extracted via Unix command line for positive selection analysis. These sequences were subsequently aligned at the codon level using PAL2NAL v14.1^61^ to ensure precise alignment for downstream selection tests. Phylogenetic trees for each single-copy orthogroup were constructed using IQ-TREE2 v2.3.6,^57^ resulting in Newick-formatted tree files. The aligned sequences and their corresponding tree files were then analyzed using the Codeml program within the PAML v 4.10.7 package^62^ to estimate log-likelihood (lnL) values and Bayes Empirical Bayes (BEB) probabilities for individual sites. Positive selection was evaluated using the Likelihood Ratio Test (LRT), with *P*-values adjusted for multiple testing using the Benjamini–Hochberg method. A significance threshold was established at padj <0.05. For analyses of both gene family evolution and positive selection, functional enrichment studies were conducted, encompassing Gene Ontology (GO) terms and Kyoto Encyclopedia of Genes and Genomes (KEGG) metabolic pathway enrichments. These analyses were performed using ShinyGO v1.1.0,^63^ with *Danio rerio* (zebrafish) selected as the reference species for functional annotation and pathway mapping. GO and KEGG enrichments were considered significant at a false discovery rate (FDR) threshold of <0.01. All heatmaps were generated utilizing the R packages pheatmap v1.0.12 and ggplot2 v3.5.1.^64^

### 2.7. Differential expression analysis of epithelial barrier-associated genes

To investigate tissue-specific expression patterns of epithelial barrier-associated genes, publicly available RNA-seq datasets from multiple tissues of *Engraulis encrasicolus* were retrieved from the NCBI Sequence Read Archive (SRA). The analyzed datasets included caudal fin (SRR22226721–SRR22226723), fin (SRR22226724–SRR22226726), gill (SRR22226727–SRR22226729), kidney (SRR4431642–SRR4431643), liver (SRR4431644–SRR4431645), ovary (SRR4431647–SRR4431649), and testis (SRR4431651–SRR4431652). Raw sequencing reads were aligned against the chromosome-scale *E. encrasicolus* reference genome using HISAT2 v2.2.1^65^ under default parameters and then indexed and sorted with Samtools v1.16.1.^66^ Gene-level read counts were generated using featureCounts v2.0.3^67^ based on the final genome annotation. Differential gene expression analysis was performed in R using DESeq2 v1.44.0.^68^ Differential expression was assessed by comparing gill tissue against all other tissues combined. Genes with an adjusted *P*-value (Benjamini–Hochberg false discovery rate, FDR) < 0.05 and an absolute log2 fold change > 1 were considered significantly differentially expressed. To characterize epithelial barrier integrity-related transcriptional responses, differentially expressed genes were assigned to 4 functional categories: tight junctions, cytokine signaling, chemokine signaling, and gap junction communication. Candidate genes were identified through Gene Ontology (GO) annotation by searching GO descriptions and functional annotations associated with each category. Genes matching the relevant GO terms were manually curated and retained for downstream analyses and visualization.

## 3. Results

### 3.1. Chromosome-level genome assembly and annotation of the *E. encrasicolus* genome

**Fig. 1.**
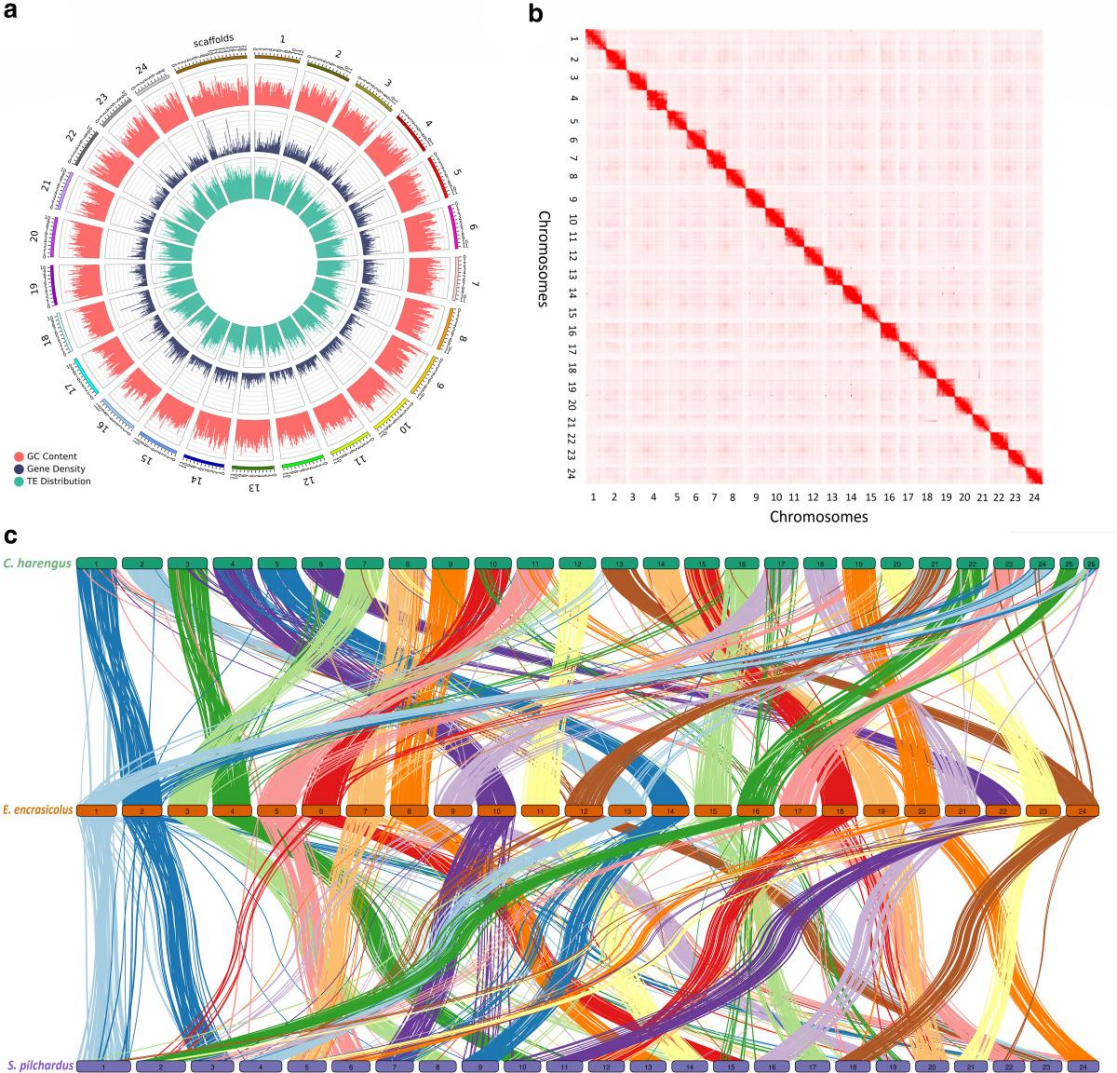
(a) Genomic features of *E. encrasicolus*: The innermost ring represents transposable element (TE) density (green). The middle ring shows gene density (blue), and the outermost ring illustrates GC content (pink). All histograms are calculated using 1-Mb windows. (b) Hi–C contact map based on the chromosome-scale assembly of the *E. encrasicolus* genome: The contact map, generated using Hi–C data aligned to the chromosome-scale assembly, is visualized as a heatmap that highlights the interaction matrices, revealing the spatial organization of the genome at the chromosomal level. (c) Syntenic relationships among 3 Clupeiformes species: *E. encrasicolus*, *C. harengus*, and *S. pilchardus*: Each colored block represents a syntenic region with homologous genes arranged in the same order across the 3 species. Aligning these regions highlights the extent of genomic conservation, reflecting their evolutionary relationships and genomic stability within the Clupeiformes order.

A chromosome-level genome sequence of *E. encrasicolus* was assembled utilizing multiple sequencing platforms. Before genome assembly, a genome sequencing survey was conducted to estimate genome size, heterozygosity, and repeat content. For this survey analysis, over 250 Gb of high-quality Illumina reads were used for *k-*mer analysis (Table S1). GenomeScope estimation revealed a haploid genome length ranging from 1.47 Gb to 1.72 Gb, with model fitness between 99.19% and 97.368% and a read error rate of 0.06% to 0.08% (using *k*-mer values of 19 and 31, respectively) (Figure S1a-b). The 19-mer size exhibited a peak model fitness of 99.19% and a minimum read error rate of 0.060%. Therefore, 19-mers were used for further analysis. Following genome survey analysis, a high-quality chromosome-level genome sequence of *E. encrasicolus* was produced using multiple technologies. A total of 161.57 Gb of PacBio CLR reads (∼110X), 178.86 Gb of stLFR reads (∼121X), and 91.14 Gb of Hi-C reads (∼62X) were used to generate this assembly (Table S2; depths based on estimated genome size). An initial assembly was generated using corrected PacBio CLR reads, resulting in a 1.69 Gb genome with a contig N50 of 973 Kb and a longest contig of 9.334 Mb. Following this, stLFR linked reads and Hi-C scaffolding were used to anchor contigs to chromosomes. After duplicate removal, the final assembly comprised 1.4 Gb of sequence placed onto 24 chromosomes (Fig. 1a). The scaffold N50 reached 56.54 Mb, with the longest scaffold measuring 60.45 Mb. The assembly had a GC content of 44.50% (Table 1). The Hi–C contact matrix showed clear chromosome territories with strong diagonal interactions across all 24 chromosomes, validating the chromosome-level assembly quality of the *E. encrasicolus* reference genome (Fig. 1b). Synteny analysis revealed chromosomal rearrangements and complex patterns of collinear blocks among 3 Clupeid species, indicating significant genomic restructuring during their evolutionary divergence (Fig. 1c). Genome completeness, assessed by quantifying universally conserved single-copy genes (BUSCO v5 with Actinopterygii_odb10 database), indicated a high-quality assembly with 96.6% completeness (Table 1).

**Table 1.** Chromosome-level genome assembly statistics for the *E. encrasicolus* (Gb: Gigabase, Mb: Megabase).

Statistic	Value
Genome size	1.4 Gb
Total ungapped length	1.4 Gb
Number of chromosomes	24
Number of organelles	0
Number of scaffolds	1,921
Scaffold N50	56.4 Mb
Scaffold L50	13
Number of contigs	4,086
Contig N50	1.4 Mb
Contig L50	246
GC percent	44.5
Genome coverage	180.0x
# of sequences >1 K (nt)	1,898 (98.8% of total number)
# of sequences >10 K (nt)	1,344 (69.9% of total number)
# of sequences >100 K (nt)	155 (8.1% of total number)
# of sequences >1 M (nt)	136 (1.9% of total number)
# of sequences >10 M (nt)	24 (1.2% of total number)
Sum length of sequences >1 M (nt)	1,363,312,605 (95.0% of total length)
Sum length of sequences >10 M (nt)	1,338,022,500 (93.2% of total length)
Total core genes	*n* = 3,640 (Actinopterygii_odb10)
Overall complete [C = S + D] BUSCO score	96.6%
Single-copy [S] orthologs	94.1%
Duplicated [D]orthologs	2.5%
Fragmented [F] genes	0.5%
Missing [M] genes	2.9%

Repetitive elements were characterized through a combination of de novo identification and homology-based annotation. In total, 3,762,132 repeat sequences spanning ∼640 Mb were identified, accounting for 44.60% of the *E. encrasicolus* genome (Table S3). Class II DNA transposons constituted the largest fraction of the repeat landscape, representing 16.00% of the genome and 35.89% of all masked bases. Within this class, CACTA (39.25%), hAT (39.48%), and TcMar (9.61%) superfamilies were the most abundant (Table S4). Class I retrotransposons were dominated by LTR elements (6.40% of the genome), among which Gypsy (48.13%), ERV (14.05%), and Copia (13.14%) were the predominant superfamilies. Among non-LTR retrotransposons, LINEs accounted for 60.57% of the combined LINE/SINE/PLE pool, followed by SINEs (30.18%) and Penelope-like elements (9.24%). Class III Helitron elements constituted a minor fraction (0.27% of the genome). Simple repeats and low-complexity regions together comprised an additional 11.15% of the genome. A substantial portion (10.04%) of the repeat content could not be classified and remained of unknown origin (Tables S3, S4; Figure S2). A comprehensive dataset comprising 53.6 Gb of clean transcriptomic sequences (NCBI BioProject: PRJNA348159) was utilized to facilitate genome annotation. Following repeat masking, gene prediction was performed using an integrated pipeline combining ab initio, homology-based, and RNA-Seq-guided transcript evidence. Our approach yielded 35,487 total gene models, with the vast majority (90.53%) successfully anchored to chromosomes. We have consolidated the specific counts and lengths for protein-coding genes, exons, introns, ncRNAs, and pseudogenes directly into Table 2. Additionally, functional annotation successfully classified most predicted mRNAs through the integration of SwissProt, GO, NCBI nonredundant (NR), and KEGG databases.

**Table 2.** Genome annotation statistics and gene prediction metrics for *E. encrasicolus* (Mb: Megabase).

Genome annotation	Count	Total length (Mb)	Percentage (%)
Total genes	35,487	828,652,844	57.73
Genes in a chromosome	32,125	799,297,693	55.69
Protein-coding genes	27,663	798,011,719	55.60
mRNAs	40,879	142,505,861	9.92
CDSs	41,019	92,889,362	6.42
Exons	315,443	87,978,957	6.13
Introns	281,608	799,115,928	55.68
Noncoding RNAs	8,968	128,886	0.008
tRNA	1,704	97,212	0.006
rRNA	16	1,857	0.0001
lncRNA	6,272	15,361	0.001
snoRNA	434	308	0.00002
snRNA	189	192	0.00001
Pseudogenes	538	7,305,939	0.5

### 3.2. Lineage-specific gene family expansions and adaptation in filter-feeding clupeid fishes

**Fig. 2.**
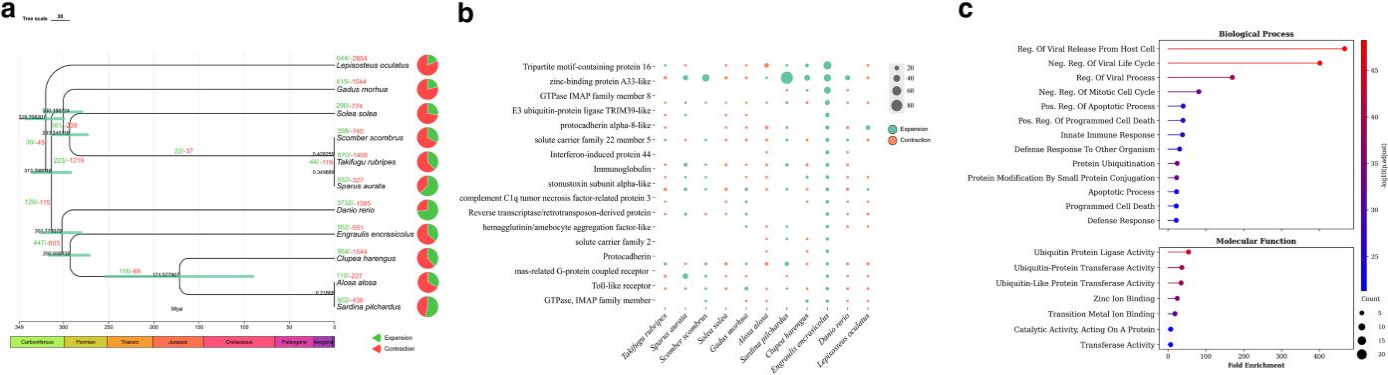
Evolutionary dynamics of gene family diversification and functional enrichment. a) A time-calibrated phylogenetic tree illustrating lineage-specific expansion (green) and contraction (red) events. Values on the branches denote the absolute number of expanded and contracted gene families, whereas pie charts summarize the relative proportional shifts within each species. b) The cross-species distribution of alterations in gene family size. Bubble volume corresponds to gene copy number, with color gradients signifying patterns of expansion or contraction. c) GO enrichment analysis of genes implicated in family size variations, categorized by BP and MF. Dot magnitude reflects the underlying gene count, color scaling represents statistical significance (−log10 adjusted *P* value), and fold enrichment denotes the effect size.

To investigate the evolutionary genomic architecture of gene families, we conducted a comparative analysis of gene family dynamics between *E. encrasicolus* and other Clupeidae members, as well as reference species with well-characterized genomes. A total of 11,755 orthogroups were identified across the compared species, of which 552 showed significant expansion and 951 exhibited contraction in *E. encrasicolus* (*P* < 0.05, CAFE5), reflecting a net contractive trend shared across the Clupeidae, as evidenced by 447 expansions and 603 contractions at the ancestral Clupeidae node (Fig. 2a). This pattern was not unique to *E. encrasicolus* but appears to be a broader characteristic of the Clupeidae, as the ancestral Clupeidae node itself showed 447 expansions against 603 contractions, suggesting that genomic contraction was already underway prior to the radiation of extant clupeoid lineages (Fig. 2a). The time-calibrated phylogeny further revealed that this contractive trend intensified along terminal Clupeidae branches, while outgroup species such as *D. rerio* displayed markedly more balanced or expansive dynamics (3,732 expanded/1,385 contracted), highlighting the evolutionary distinctiveness of the Clupeidae gene family landscape. The cross-species distribution of gene family size alterations is illustrated in Fig. 2b, where bubble volume reflects gene copy number and color gradient distinguishes expansion (green) from contraction (orange) across all 11 compared taxa. This visualization reveals that several functionally important gene families exhibit prominent copy number variation across the compared species. Among these, zinc-binding protein A33-like and GTPase IMAP family member 8 stand out as showing particularly large copy number differences, with *E. encrasicolus* displaying notable expansion relative to non-Clupeidae species. Additional families showing cross-species variation include tripartite motif-containing protein 16 (TRIM16), interferon-induced protein 44, immunoglobulin, Toll-like receptor, protocadherin, and solute carrier families, suggesting that immune surveillance and membrane transport gene repertoires have been subject to recurrent and lineage-specific copy number evolution across teleosts.

To characterize the functional roles of the observed expansions, GO enrichment analysis was performed on *E. encrasicolus*-specific expanded gene families, revealing a coherent biological signal dominated by antiviral immunity, innate defense, and regulated cell death (Fig. 2c). The most significantly enriched biological processes were regulation of viral release from host cells and negative regulation of viral life cycle (>400-fold enrichment, FDR<10^−6^), followed by regulation of viral processes (∼175-fold), negative regulation of the mitotic cell cycle, and positive regulation of apoptosis and programmed cell death, collectively suggesting that expanded gene families in *E. encrasicolus* are disproportionately weighted toward pathogen defense and the regulation of cellular fate under immune challenge. At the molecular function level, ubiquitin protein ligase, ubiquitin-protein transferase, and ubiquitin-like protein transferase activities showed the strongest enrichment (∼50-fold, FDR<10^−6^), alongside zinc ion and transition metal ion binding, consistent with the species-specific expansion of zinc-finger immune proteins such as TRIM16 and zinc-binding protein A33-like. Taken together, these results indicating that immune gene family expansion in antiviral restriction and ubiquitin-mediated protein turnover represents a prominent evolutionary signature in *E. encrasicolus*, potentially associated with adaptation to its pathogen-rich pelagic environment.

**Fig. 3.**
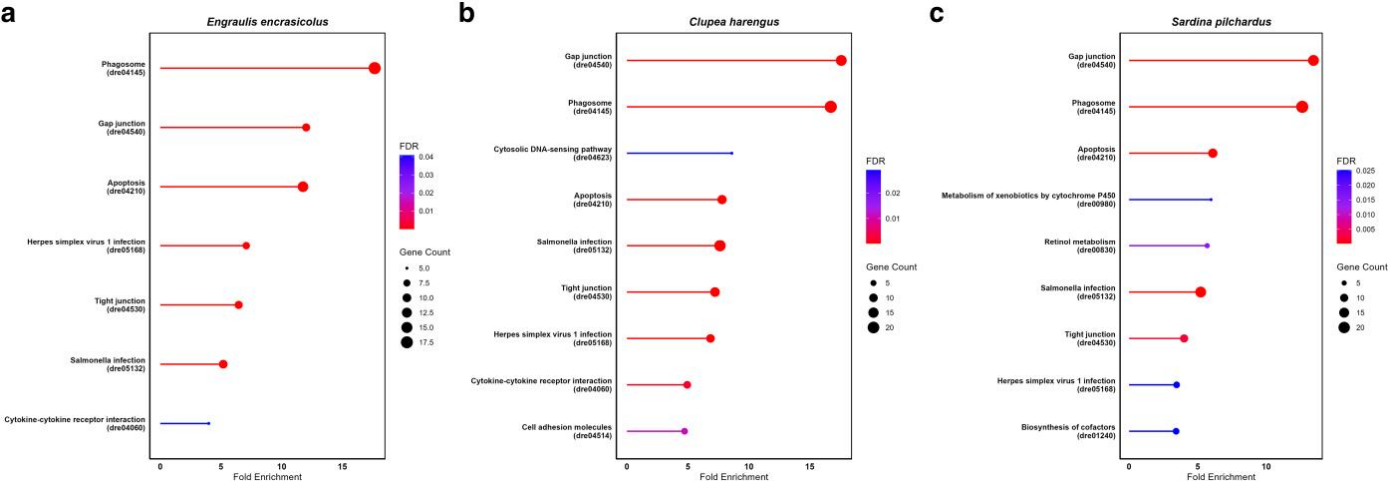
KEGG pathway enrichment analysis of expanded gene families identified by CAFE. Lollipop plots represent significantly enriched pathways for gene families that underwent expansion in (a) *E. encrasicolus*, (b) *C. harengus*, and (c) *S. pilchardus*. The *x*-axis indicates the fold enrichment score for each pathway. The size of the bubbles reflects the gene count (total number of genes within the expanded families for that category), and the color scale represents the statistical significance via the FDR.

KEGG pathway enrichment analysis of expanded gene families across the 3 Clupeidae species revealed both shared and species-specific patterns (Fig. 3). Phagosome (dre04145) and Gap junction (dre04540) pathways were significantly enriched in all 3 species, with phagosome showing the highest fold enrichment in *E. encrasicolus* (∼17-fold) and gap junction ranking highest in both *C. harengus* and *S. pilchardus* (∼17-fold and ∼13-fold, respectively), indicating that immune phagocytic function and intercellular communication have been subject to recurrent gene family expansion across Clupeidae. Apoptosis (dre04210), Herpes simplex virus 1 infection (dre05168), *Salmonella* infection (dre05132), and Tight junction (dre04530) were also consistently enriched across all 3 species, reinforcing the pattern of pathogen-driven expansion identified in the GO analysis. Species-specific enrichments provided additional resolution: *E. encrasicolus* uniquely showed enrichment in cytokine–cytokine receptor interaction (dre04060); *C. harengus* exhibited enrichment in the cytosolic DNA-sensing pathway (dre04623) and cell adhesion molecules (dre04514); and *S. pilchardus* was uniquely enriched in metabolism of xenobiotics by cytochrome P450 (dre00980) and retinol metabolism (dre00830), pointing to species-specific divergence in detoxification and metabolic pathways. Collectively, the KEGG results corroborate and extend the GO enrichment findings, demonstrating that gene family expansion in Clupeidae has acted preferentially on immune defense, pathogen recognition, and cell death regulation, with additional species-level specializations likely reflecting divergent ecological pressures.

### 3.3. Detecting signatures of genome-wide positive selection in Clupeid genomes

**Fig. 4.**
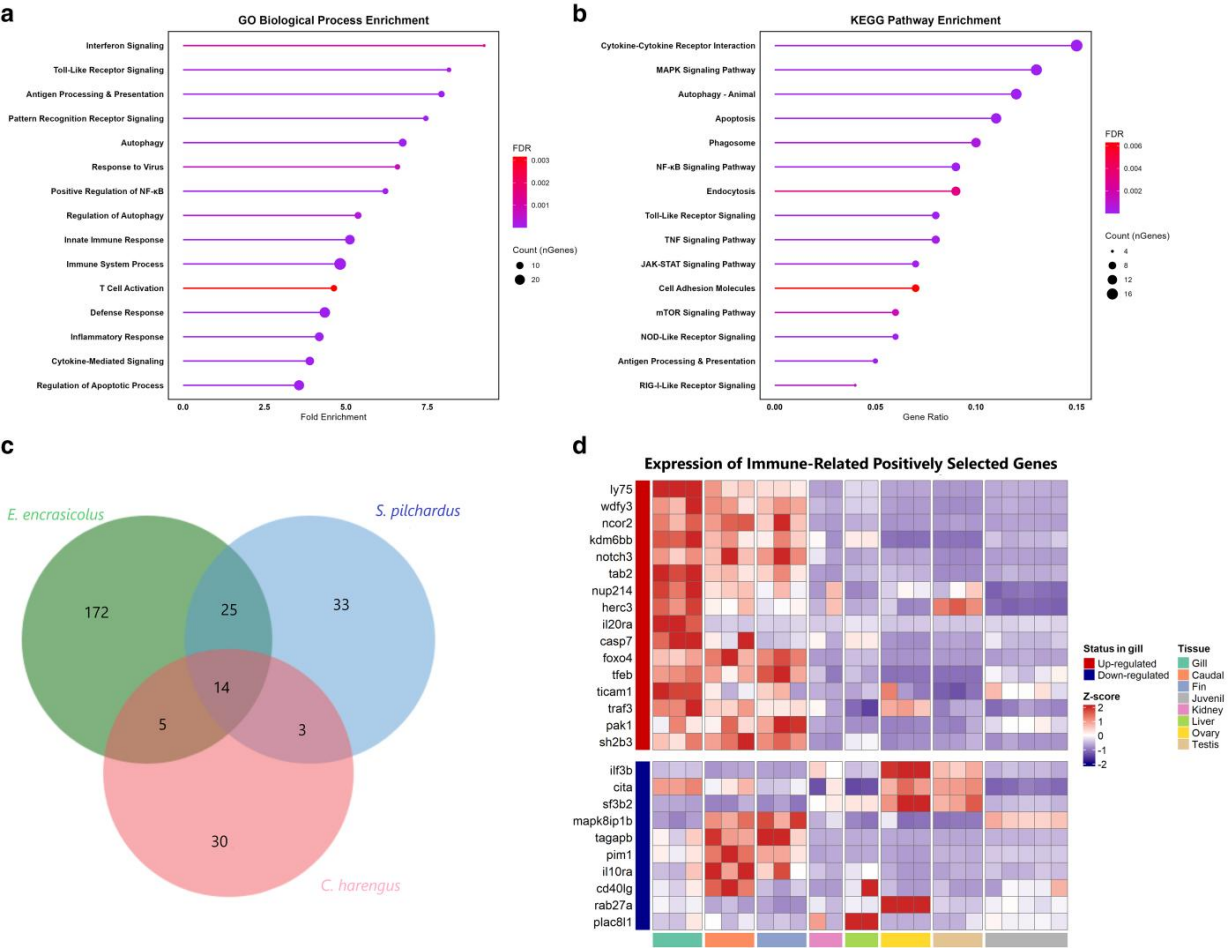
Functional landscape and expression of PSGs in *E. encrasicolus*. Lollipop plots of enriched (a) GO BP and (b) KEGG pathways; dot size and color represent gene count and FDR, respectively. c) Venn diagram comparing orthologous PSGs among *E. encrasicolus, S. pilchardus*, and *C. harengus*. d) Heatmap of immune-related PSGs across tissues, showing expression status in gill (up/down-regulated).

To investigate the potential mechanism of molecular adaptation underlying the characteristic filter-feeding behavior of Clupeid fishes, we performed a genome-wide scan for signatures of positive selection using a comparative evolutionary framework. We specifically identified immune-related loci that exhibit selection signatures unique to the Clupeid lineage, occurring both along the ancestral branches and within the clade. We identified 216 PSGs on the *E. encrasicolus* branch, 75 PSGs in *S. pilchardus*, 52 PSGs in *C. harengus*, and 299 PSGs along the ancestral Clupeidae lineage (Dataset 2). Venn diagram analysis across the 3 species revealed 14 genes under convergent positive selection in all 3 species, including the immune-related genes *nup214*, *tab2*, *cita*, *ilf3b*, and *ncor2* (Fig. 4c). *E. encrasicolus* harbored the largest number of species-specific PSGs (172), followed by *S. pilchardus* (33) and *C. harengus* (30), while pairwise overlaps were highest between *E. encrasicolus* and *S. pilchardus* (25 shared genes). A further 40 genes were under positive selection in both the *E. encrasicolus* branch and the ancestral Clupeidae lineage, indicating sustained selective pressure across the clade.

Functional enrichment analysis of PSGs revealed a pronounced overrepresentation of immune-related biological processes and pathways. GO BP enrichment identified terms spanning the full spectrum of immune defense, with the highest fold enrichment observed for “Interferon Signaling,” “Toll-like Receptor Signaling,” “Antigen Processing and Presentation,” and “Pattern Recognition Receptor Signaling,” alongside broader categories including “Autophagy,” “Response to Virus,” “Positive Regulation of NF-κB,” “Immune System Process,” “Defense Response,” and “T Cell Activation” (Fig. 4a). KEGG pathway enrichment corroborated these findings, with the most significantly enriched pathways being “Cytokine–Cytokine Receptor Interaction,” “MAPK Signaling Pathway,” “Autophagy—Animal,” “Apoptosis,” “Phagosome,” “NF-κB Signaling Pathway,” “Toll-like Receptor Signaling,” and “RIG-I-like Receptor Signaling” (Fig. 4b). These results collectively indicate that immune defense, pathogen recognition, and intracellular clearance pathways have been the primary targets of positive selection in Clupeid genomes.

A detailed functional annotation of immune-related PSGs identified 81 genes distributed across 15 functional categories (Dataset 3). Antiviral and innate immune signaling constituted the largest category (15 genes), including MAPK cascade components (*mapk8ip1b*, *mapk8ip2*, *mapk10*, *mapk13*, *mapkapk5*, *map2k1*, *map3k2*), TLR-adaptor molecules *ticam1* (TRIF) and *traf3*, the NF-κB signaling mediator *tab2*, the viral RNA-binding factor *ilf3b*, and the E3 ubiquitin ligase *herc3*. Epigenetic immune regulation was the second-largest immune category (8 genes), with positive selection detected in histone demethylases *kdm6bb*, *kdm5a*, and *kdm5c*, the histone methyltransferases *ehmt2* (G9a), *ash1 l*, and *dot1l*, the DNA methyltransferase *dnmt1*, and the transcriptional corepressor *ncor2* (SMRT), suggesting that chromatin-level regulation of immune gene expression has been a recurrent target of adaptive evolution. Ubiquitin-mediated immunity was equally prominent (8 genes), encompassing TRIM-family E3 ligases (*trim32*, *trim36*), RING-finger proteins (*rnf19b*/NKLAM, *rnf169*), and deubiquitinases (*usp47*, *otud7b*/Cezanne), pointing to extensive diversification of the ubiquitin-proteasome system in pathogen defense.

Among the genes under convergent selection, a striking commonality was observed in autophagy and nuclear transport pathways. The autophagy master regulator *tfeb* and the selective autophagy adaptor *wdfy3* (ALFY) were positively selected in both the *E. encrasicolus* branch and the ancestral Clupeidae lineage, alongside additional autophagy components *rb1cc1* (FIP200) and *ulk3*, pointing to sustained selective pressure on the xenophagy pathway for clearance of intracellular bacteria and viruses. The nuclear pore complex component *nup214*—the only gene under positive selection across all 4 branches (all 3 species and the lineage)—is a known viral docking target mediating nuclear import and export of viral genomes, suggesting a persistent evolutionary arms race at the host–pathogen interface. Branch-specific analysis revealed that *E. encrasicolus* showed unique adaptation in antigen presentation (*cita*/CIITA, the master regulator of MHC class II genes), DNA damage-associated immune signaling (*atm*, *fancd2*), mucosal barrier defense (*lsr*, *keap1b*), and multiple epigenetic regulators (*ehmt2*, *dnmt1*, *dot1l*). The Clupeidae lineage exhibited selection in adaptive immunity regulators including *cd40lg* (essential for T-cell–B-cell interaction), *tagapb*, *clnk*, and *sh2b3* (LNK), as well as complement component *c5*, the executioner caspase *casp7*, and the anti-inflammatory cytokine receptor *il10ra*. Of the 81 immune PSGs, 25 were classified as antiviral and antibacterial, 21 as primarily antiviral, 14 as antibacterial, and 21 as immune regulatory, reflecting broad diversification of both pathogen-sensing and immune-modulatory mechanisms.

Tissue-specific expression profiling of immune-related PSGs in *E. encrasicolus* using RNA-Seq data revealed a conspicuous pattern of gill-biased expression (Fig. 4d). Of the 26 immune-related PSGs examined, 16 exhibited markedly elevated expression in gill tissue relative to other tissues. The most prominent gill up-regulated genes included *ly75* (CD205; dendritic cell antigen receptor), *wdfy3* (selective autophagy adaptor), *ncor2* (nuclear receptor corepressor 2), *kdm6bb* (histone demethylase involved in trained immunity), *tab2* (NF-κB signaling adaptor), *nup214* (nuclear pore complex), *il20ra* (epithelial interleukin receptor), *herc3*, *ticam1* (TRIF), *traf3*, *casp7*, *notch3*, *foxo4*, *tfeb*, *pak1*, and *sh2b3*. This gill-centric expression pattern is consistent with the functional role of branchial tissue as a primary mucosal immune barrier in teleost fishes, where constant exposure to waterborne pathogens imposes strong selective demands on antigen recognition, intracellular clearance, and epithelial defense pathways. The co-occurrence of positive selection and elevated gill expression in genes spanning autophagy (*tfeb*, *wdfy3*), innate immune signaling (*ticam1*, *traf3*, *tab2*), antigen presentation (*ly75*), epigenetic regulation (*kdm6bb*), and nuclear transport (*nup214*) suggests that gill-associated immune functions have been a major evolutionary target in Clupeid fishes (Fig. 4d; Dataset 3).

### 3.4. Differential expression of epithelial barrier integrity-associated gene families in *E. encrasicolus*

**Fig. 5.**
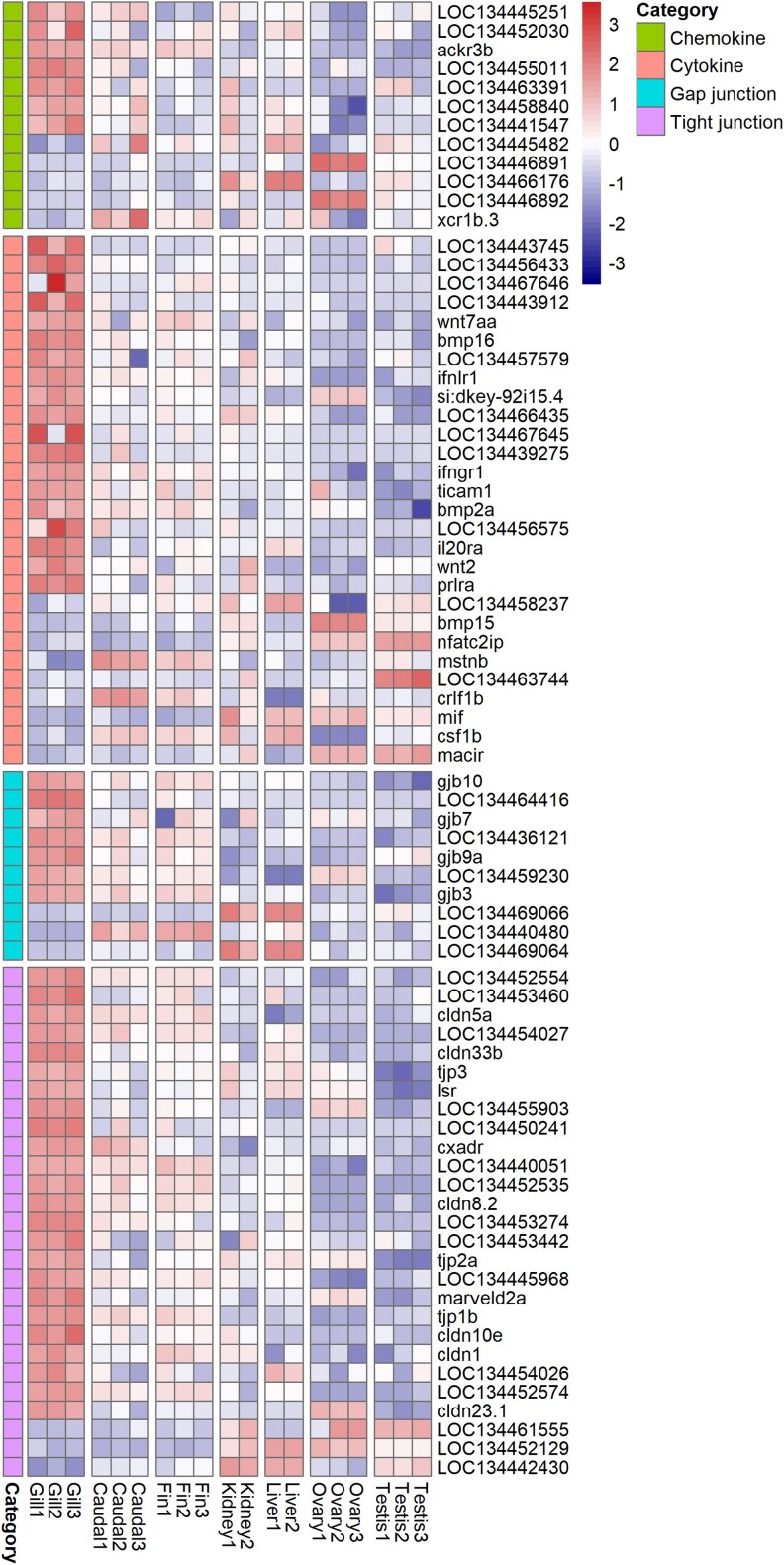
Heatmap of differentially expressed genes associated with epithelial barrier integrity across multiple tissues in *E. encrasicolus*. Rows represent individual genes grouped by functional category (chemokine, green; cytokine, red; gap junction, cyan; tight junction, purple), and columns represent biological replicates from 8 tissues (gill, caudal fin, fin, kidney, liver, ovary, and testis). Color intensity reflects row-scaled normalized expression values (*z*-score), with red indicating higher and blue indicating lower relative expression. Gene identifiers are shown on the right axis.

To investigate the molecular basis of epithelial barrier integrity in *E. encrasicolus*, we examined the differential expression of genes associated with 4 key functional categories: tight junctions, cytokine signaling, chemokine signaling, and gap junction communication. A total of 75 DEGs were identified across these categories, of which 56 were upregulated and 19 were downregulated (Fig. 5; Table S5). Tight junction-associated genes represented the most extensively modulated category, with 22 of 25 DEGs showing upregulation (Fig. 5). The upregulated set was dominated by claudin family members (claudin-1, -3-like, -4-like [4 paralogs], -5a, -7, -8-like, -8.2, -9-like, -10e, -23.1, and -33b), scaffolding proteins tjp1b (ZO-1), tjp2a (ZO-2), tjp3 (ZO-3), tricellular junction components lsr and marveld2a (tricellulin), JAM-A-like, and CXADR, while only 3 claudins (claudin-14-like, -15-like, and -18-like) were downregulated, indicating broad junctional reinforcement across both bicellular and tricellular tight junction complexes. Notably, tight junction genes showed the highest expression levels in gill tissue relative to other tissues examined (Fig. 5). Cytokine signaling genes constituted the largest DEG category (28 genes; 20 up, 8 down), with upregulated genes encoding multiple TNFRSF14 (HVEM) paralogs, butyrophilin subfamily 1 member A1-like, interferon receptors (*ifngr1*, *ifnlr1*), *il20ra*, *prlra*, and the TLR adaptor TICAM1 (TRIF), as well as developmental signals (bmp2a, bmp16, wnt7aa, wnt2), while genes encoding macrophage migration inhibitory factor (mif), colony stimulating factor 1b (csf1b), and macrophage immunometabolism regulator (macir) were downregulated, suggesting a shift from macrophage-centric inflammation toward receptor-mediated epithelial immune surveillance. Chemokine-associated genes (12 DEGs; 7 up, 5 down) showed upregulation of CC-motif ligands (CCL19-like, CCL20-like), monocyte chemotactic protein 1B-like, and ackr3b, alongside downregulation of CXCL11-6-like, CCL5-like, and CCL26-like, indicating selective remodeling of chemokine signaling axes. Gap junction genes (10 DEGs; 7 up, 3 down) showed predominantly increased expression of connexin beta family members (*gjb3*, *gjb7*, *gjb9a*, *gjb10*) and 3 additional connexin paralogs (Table S5). These expression patterns point to a coordinated transcriptional program of tight junction reinforcement, cytokine and chemokine receptor diversification, and enhanced gap junction communication in *E. encrasicolus*, with gill tissue displaying the most pronounced expression across all 4 functional categories.

## 4. Discussion

The chromosome-scale assembly of *E. encrasicolus* reported here (1.4 Gb; 24 chromosomes; scaffold N50 56.4 Mb; BUSCO 96.6%; GC 44.5%) represents the first high-resolution reference genome for the genus *Engraulis* in the Mediterranean–Atlantic range. With 27,663 protein-coding genes and 44.6% repetitive content, the assembly is comparable in gene count and completeness to related clupeiforms but reveals a striking genome-size disparity within the order. The Atlantic herring spans only 726 Mb across 26 chromosomes,^69^ the European sardine *S. pilchardus* 869 Mb on 24 chromosomes,^70^ the tapertail anchovy *Coilia nasus* 851 Mb,^71^ and the dotted gizzard shad *Konosirus punctatus* approximately 800 Mb,^72^ while the American shad *Alosa sapidissima* falls within a comparable range.^73^ Against this background, the near doubling of genome size observed in *E. encrasicolus* and its congener *E. japonicus* (1.4 Gb)^74^ is unlikely to reflect polyploidy, given that 24 chromosomes and low BUSCO duplication rates are consistent with a standard diploid clupeiform karyotype. The variation in genome size is best explained by the lineage-specific expansion of transposable elements.^75–77^ Unlike the retrotransposon-heavy genomes of sardines and herring, the anchovy genome is characterized by a high density of Class II DNA transposons, which account for roughly 36% of its repetitive DNA. This suggests a recent burst of DNA transposon activity unique to the *Engraulis* lineage. Beyond simply increasing genome size, this TE-driven restructuring provided the structural and regulatory foundation for further evolution, specifically facilitating the expansion of immune-related genes.

Analysis of gene-family dynamics across 11,755 orthogroups reveals an evolutionary paradox when viewed within this repeat-dense genomic landscape. While the Clupeidae exhibit a prevailing trend toward gene-family contraction—observed both at the ancestral node (447 expanded vs. 603 contracted) and within *E. encrasicolus* (552 expanded vs. 951 contracted)—this global loss is punctuated by the significant expansion of innate-immune gene families. Specifically, the expanded orthogroups in the *E. encrasicolus* are characterized by an enrichment of TRIM16 and other tripartite-motif paralogues, GTPase IMAP member 8, zinc-binding protein A33-like, interferon-induced protein 44 (IFI44), immunoglobulin loci, and various Toll-like receptor families. GO enrichment identifies regulation of viral release from host cells and negative regulation of viral life cycle as the most over-represented categories (>400-fold enrichment), alongside ubiquitin-mediated protein turnover, while KEGG pathways shared by all 3 clupeid species examined (phagosome, apoptosis, gap junction, tight junction, Herpes simplex virus 1 infection, and *Salmonella* infection) point to a coordinated innate-immune and epithelial-barrier signature. These findings echo, with a distinctly clupeid accent, patterns documented in other teleost lineages: the zebrafish TRIM repertoire exceeds 200 members,^78,79^ and copy-number variation at herring immune loci^80^ demonstrates that clupeid immune gene architecture is intrinsically plastic. The comparison with Atlantic cod provides a particularly instructive counterpoint, since *Gadus morhua* lost MHC class II entirely and compensated through massive expansions of MHC-I and TLR families,^81–83^ whereas *E. encrasicolus* appears to retain functional MHC-II while amplifying antiviral restriction factors and E3 ubiquitin ligase machinery. We interpret this expansion pattern as a genomic signature consistent with the filter-feeding ecology characteristic of engraulids and clupeids: nonselective ram ventilation through densely packed gill rakers exposes the branchial epithelium to an unusually high and continuous pathogen inoculum, creating persistent selective pressure on the genes that detect, restrict, and clear waterborne microbes. Under this ecological framework, one would expect the expanded genes to show evidence of adaptive evolution (positive selection). Furthermore, their activity should be concentrated in the gills—the organ most directly impacted by these specific environmental stressors.

The positive selection analyses presented here provide exactly this predicted signal. Of the 216 PSGs detected in *E. encrasicolus*, compared with 75 in *S. pilchardus*, 52 in *C. harengus*, and 299 on the ancestral Clupeidae lineage, 14 are convergently selected in all 3 species (including *nup214*, *tab2*, *cita*, *ilf3b*, and *ncor2*), and 81 map to 15 immune functional categories. Four interlocking modules dominate this landscape: antiviral and innate signaling (MAPK cascade components, *ticam1*/TRIF, *traf3*, *tab2*, NF-κB mediators), epigenetic immune regulation (*kdm6bb*, *kdm5a*, *ehmt2*, *dnmt1*, *ncor2*), ubiquitin-mediated immunity (*trim32*, *trim36*, *rnf19b*, *usp47*, *otud7b*), and selective autophagy (*tfeb*, *wdfy3*/ALFY, *rb1cc1*/FIP200, *ulk3*). Selection on *nup214* across all 4 tested branches is particularly telling, since the nuclear pore complex is a canonical viral docking station and convergent signatures at this locus point to a deep, clade-wide arms race at the nuclear envelope. Equally noteworthy is the *E. encrasicolus*-specific selection on *cita*/CIITA, the master transactivator of MHC class II,^84^ a finding that reinforces our earlier inference that clupeids preserve and actively remodel their MHC-II axis rather than abandoning it as cod have done. The epigenetic PSGs connect the anchovy signal to the rapidly expanding literature on trained immunity in teleosts, where histone-modifying enzymes encode an adaptive-like innate memory.^85,86^ The co-selection of autophagy regulators alongside ubiquitin machinery further suggests coupled remodeling of cargo recognition and lysosomal delivery systems for intracellular pathogen clearance. Critically, 16 of the 26 testable immune PSGs show gill-biased expression, thereby anchoring these evolutionary signatures to the anatomical site where selective pressures are expected to be strongest. This organ-level convergence between expansion and selection data raises the question of whether the gill transcriptome itself bears additional, expression-level hallmarks of barrier reinforcement.

The differential expression analysis of gill tissue provides precisely such evidence, offering a third, mechanistically explicit layer of support. Of 75 barrier-associated DEGs, 25 encode tight-junction components (22 upregulated), 28 are cytokines (20 up), 12 chemokines (7 up), and 10 gap-junction genes (7 up), with gill tissue displaying the highest expression across every functional category. At least 11 claudin paralogues are transcriptionally activated, including claudin-1, multiple claudin-3 and claudin-4 isoforms, claudin-5a, -7, -8, -9, -10e, -23.1, and -33b, alongside the zonula-occludens scaffolding proteins ZO-1 (*tjp1b*), ZO-2 (*tjp2a*), and ZO-3 (*tjp3*), and the tricellular junction components *lsr* and *marveld2a* (tricellulin). Such diversity is consistent with the long-recognized expansion of claudin genes in teleosts, exemplified by the approximately 56 paralogues in *Takifugu rubripes* compared with 19 in humans,^87^ and with the functional evidence that claudin isoform composition sets both paracellular permeability and pathogen exclusion in gill ionocytes and pavement cells.^88,89^ The parallel upregulation of 7 connexin-β gap-junction genes adds a layer of intercellular coordination, likely propagating danger signals and metabolites across the lamellar epithelium, recalling the expanded connexin repertoires previously documented in zebrafish (39 genes vs. 21 in mammals).^90,91^ The cytokine expression profile is equally informative: multiple TNFRSF14 (HVEM) paralogues, interferon receptors *ifngr1* and *ifnlr1*, IL-20Rα, and the TLR adaptor *ticam1*/TRIF are upregulated, while macrophage-centric factors (MIF, CSF1b, MACIR) are downregulated, suggesting a shift from systemic macrophage-driven surveillance toward epithelial-intrinsic, interferon-gated antiviral defence. These transcriptional patterns align the anchovy gill with the emerging concept of gill-associated lymphoid tissue (GIALT) as a bona fide mucosal inductive site, where resident and recruited immune cells coordinate with epithelial barrier components to mount rapid, localized responses to waterborne pathogens.^92–94^ When viewed alongside the gene family and positive selection findings, these expression data complete a coherent, multilayered picture of branchial immune architecture in a filter-feeding teleost.

Expansions of TRIM, IFI44, GIMAP8, and TLR families supply the molecular pattern-recognition and restriction-factor substrate; positive selection tunes innate signaling, epigenetic memory, ubiquitin-mediated clearance, and xenophagy within that substrate; and gill-enriched expression of claudins, ZO scaffolds, and interferon receptors operationalizes these capacities at the organism–water interface. We hypothesize that continuous particulate filter-feeding through closely spaced gill rakers may act as a selective pressure contributing to this integrated signature, an interpretation consistent with the shared KEGG enrichments across anchovy, sardine, and herring and with the gill-biased expression of more than 60% of immune PSGs. Viewed comparatively, clupeiform evolution represents a complementary rather than parallel solution to that described for Atlantic cod: where cod discarded MHC class II and re-weighted its toolkit toward MHC-I and expanded TLRs,^81–83^ clupeids retained MHC-II, remodeled *cita*-dependent transactivation, and amplified cell-intrinsic antiviral and ubiquitin–autophagy axes. The translational implications merit consideration: small pelagic fisheries face mounting pressure from marine heatwaves and emerging pathogens, and the comparative immune-gene maps presented here furnish candidate markers for disease-resistance breeding in clupeiform aquaculture.^95^ While the multiplatform strategy used here (PacBio CLR, stLFR, and Hi-C) yielded a highly complete, chromosome-scale assembly (BUSCO 96.6%, scaffold N50 56.4 Mb) sufficient for our comparative analyses, we acknowledge the inherent limitations of the technologies available at the time. PacBio CLR reads carry higher error rates than modern HiFi (CCS) reads, and our pipeline predates recent diploid-aware, near-gapless algorithms such as *hifiasm*^96^ or *verkko*.^97^ Consequently, highly repetitive regions like telomeres, centromeres, and transposon-dense regions flanking immune loci are likely to remain incompletely resolved. Furthermore, relying on a single-individual reference genome restricts our ability to capture broader genetic diversity. Future efforts utilizing T2T-grade sequencing and updated assembly algorithms will be necessary to precisely characterize structural variation at these expanded loci. Looking ahead, population-scale resequencing and pan-genome analyses will be essential to quantify copy-number variation across wild anchovy populations, alongside single-cell transcriptomics to map the gill GIALT compartments.

In summary, by integrating a chromosome-scale *E. encrasicolus* reference with comparative orthology, selection scans, and gill transcriptomics, we propose that filter-feeding clupeiforms harbor a gill-centric immune adaptive signature potentially facilitated by transposon-fueled genome remodeling, expanded antiviral and ubiquitin–autophagy machinery, and a reinforced claudin-based epithelial barrier, representing a clupeid-specific evolutionary configuration that complements the immune architectures previously characterized in other major teleost lineages.

## Supplementary Material

dsag009_Supplementary_Data

## Data Availability

The sequencing data generated in this study, including Illumina PE, Hi-C, stLFR, and PacBio CLR reads, have been deposited in the Sequence Read Archive (SRA) of the National Center for Biotechnology Information (NCBI) under BioProject accession PRJNA348159. The specific SRA accession numbers for the libraries are SRR26346004 through SRR26346009 (Illumina short reads), SRR26346011 (Hi-C), SRR26346012 (stLFR), and SRR26346013 (PacBio CLR). The de novo chromosome-scale genome assembly (IST_EnEncr_1.0) has been deposited at NCBI/GenBank under the accession GCF_034702125.1 and is publicly accessible at https://ftp.ncbi.nlm.nih.gov/genomes/all/GCF/034/702/125/GCF_034702125.1_IST_EnEncr_1.0/.
