# Supplementary material for: Chromosome-scale genome of European anchovy (Engraulis encrasicolus L., 1758) and comparative genomics uncover clupeid-specific immune gene expansions
Source: DNA Res. 2026 Jul 15;33(4):dsag009. doi: 10.1093/dnares/dsag009 (PMC13403563; doi:10.1093/dnares/dsag009)
Supplement: dsag009_Supplementary_Data [file dsag009_supplementary_data.zip › Supplementary_Files_revised.pdf]

Supplementary Information for

Chromosome-scale genome of European anchovy (*Engraulis encrasicolus* L., 1758) and comparative genomics uncover clupeid-specific immune gene expansions

Tuana Öğretici[1], Selahattin Barış Çay[1], Onur Obut[1], Mehmet Ali Balcı[1], Yusuf Ulaş Çınar[1], Pınar Akbaba[1], Yasemin Aydın[2], Banu Orta Yılmaz[2], Nazlı Kasapoğlu[3], Şirin Firidin[3], Fatih Dikmen[2], Cem Dalyan[2], Taner Yıldız[4], Esma Gamze Aksel[5], Gökmen Zararsız[6,7], İlhan Aydın[8], Yakup Bakır[9], Melike Erkan[1], Vahap Eldem[2]*

[1]Institute of Graduate Studies in Sciences, Istanbul University, Istanbul, 34134, Türkiye

[2]Department of Biology, Istanbul University, İstanbul, 34134, Türkiye

[3]Central Fisheries Research Institute, Trabzon, 61290, Türkiye

[4]Department of Fisheries Technology and Management, Faculty of Aquatic Sciences, Istanbul University, Istanbul, 34134, Türkiye

[5]Department of Genetics, Faculty of Veterinary Medicine, Erciyes University, Kayseri, 38280, Türkiye

[6]Department of Biostatistics, Erciyes University, Kayseri, Türkiye

[7]Drug Application and Research Center (ERFARMA), Erciyes University, Kayseri, Türkiye

[8]General Directorate of Fisheries and Aquaculture, Ankara, 06800, Türkiye

[9]Department of Medical Laboratory Techniques, Vocational School of Health Services, Atlas University, Istanbul 34403, Türkiye

*Corresponding author

Email: vahap.eldem@istanbul.edu.tr

Webpage: https://avesis.istanbul.edu.tr/vahap.eldem

Office Phone: +90 212 455 5700 (Extension: 15086)

This PDF file includes:

1. Supplementary Tables (SX to SY)

2. Supplementary Figures (SX to SY)

Supplementary Tables

Table S1. The detailed information on sequencing data of *E. encrasicolus* genome sequencing survey.

**Libraries**	**Total number of clean reads**	**Clean data (Gb)**	**SRA Accession (Submitted in this study)**	**Read Length (bp)**
Anchovy_Illumina_PE1_1.fastq.gz	453,957,919	44.6		101
Anchovy_Illumina_PE1_2.fastq.gz	453,957,919	44.6	SRR26346009	
Anchovy_Illumina_PE2_1.fastq.gz	71,889,984	7.24		101
Anchovy_Illumina_PE2_2.fastq.gz	71,889,984	7.24	SRR26346004	
Anchovy_Illumina_180_1.fastq.gz	318,877,344	34.9		101
Anchovy_Illumina_180_2.fastq.gz	318,877,344	34.9	SRR26346008	
Anchovy_Illumina_400_1.fastq.gz	270,410,805	29.6		101
Anchovy_Illumina_400_2.fastq.gz	270,410,805	29.6	SRR26346007	
Anchovy_Illumina_400A_1.fastq.gz	31,578,442	3.45		101
Anchovy_Illumina_400A_2.fastq.gz	31,578,442	3.45	SRR26346006	
Anchovy_Illumina_400B_1.fastq.gz	50,687,241	5.55		101
Anchovy_Illumina_400B_2.fastq.gz	50,687,241	5.55	SRR26346005	
Total	**2,394,803,470**	**250.68**		

Table S2. The summary of sequence data used for *de novo* genome assembly (*The N50 of read length was calculated only for PacBio CLR reads).

**Libraries**	**Total number of clean reads**	**Clean data (Gb)**	**SRA Accession (Submitted in this study)**	**Read Length (bp)/N50***
European_Anchovy_Hi-C_1.fastq.gz	303,817,445	45.57		150
European_Anchovy_Hi-C_1.fastq.gz	303,817,445	45.57	SRR26346011	
European_Anchovy_stLFR_1.fastq.gz	893,819,568	89.38		100
European_Anchovy_stLFR_2.fastq.gz	893,819,568	89.38	SRR26346012	
European_Anchovy_Pac_CLR.fastq.gz	14,140,133	161.57	SRR26346013	16,817

Table S3. Distribution and classification of repetitive elements in the *E. encrasicolus* genome assembly.

TE classification	Count	Length (bp)	Percentage of Genome (%)	Percentage of Masked Bases (%)
**DNA**	701948	229724901	16.00	35.89
**LTR**	627503	91886051	6.40	14.35
**LINE**	22984	6230366	0.43	0.97
**SINE**	23678	3104604	0.22	0.48
**Penelope**	2537	951112	0.07	0.15
**Helitron**	13900	3805588	0.27	0.06
**DIRS**	1111	463216	0.03	0.07
**Unknown**	499749	144098916	10.04	22.50
**Others**	1868722	159929104	11.14	24.98
Total Repeats	3762132	640193858	44.60	100

Table S4. A detailed annotation and classification of TEs identified in *E. encrasicolus*.

Class	Subclass	Superfamily	Count	Total Length (Mb)	Percentage of Genome (%)	Percentage of Repeats (%)
**Class I**	**Unknown**		7539	5559209	0.39	0.86
	**LTR**	**Unknown**	155224	15996760	1.11	2.50
		**BEL**	15250	6680329	0.47	1.04
		**Copia**	134875	12073105	0.84	1.89
		**ERV**	140665	12910720	0.90	2.01
		**Gypsy**	181489	44225137	3.08	6.09
	**nLTR**	**Unknown**	3885	666197	0.05	0.1
		**DIRS**	1111	463216	0.03	0.07
		**LINE**	22984	6230366	0.43	0.97
		**PLE**	2537	951112	0.07	0.15
		**SINE**	23678	3104604	0.22	0.48
**Class II**	**DNA**	**CACTA**	466233	90185217	6.28	14.09
		**Harbinger**	64194	15254645	1.06	2.38
		**hAT**	18998	90698407	6.32	12.88
		**Mutator**	41697	8832805	0.62	1.25
		**PiggyBac**	9048	2685362	0.19	0.38
		**TcMar**	101778	22068465	1.54	3.13
	**MITE**		34995	4317730	0.30	0.61
	**nMITE**		275747	59579445	4.15	8.46
**Class III**	**Helitron**		13900	3805588	0.27	0.54
**Unknown**			177583	73976335	5.15	19.59
**Simple Repeats**			1749209	149906445	10.45	21.29
**Low complexity regions**			119513	10022659	0.70	1.42
**TOTAL**			3762132	640193858	44.61	100

Table S5. Differentially expressed genes associated with epithelial barrier integrity in *E. encrasicolus*, classified into four functional categories: tight junction, cytokine, chemokine, and gap junction. Up to three representative GO terms (ID and abbreviated description) are listed per gene.

Gene	Status	Protein Name	Category	GO Terms
**LOC134452554**	Up	claudin-4-like	Tight Junction	GO:0070830 (TJ assembly); GO:0005198 (structural); GO:0007155 (cell adhesion)
**LOC134453460**	Up	claudin-8-like	Tight Junction	GO:0070830 (TJ assembly); GO:0005198 (structural); GO:0007155 (cell adhesion)
**cldn5a**	Up	claudin 5a	Tight Junction	GO:0070830 (TJ assembly); GO:0005198 (structural); GO:0007155 (cell adhesion)
**LOC134454027**	Up	claudin-4-like	Tight Junction	GO:0070830 (TJ assembly); GO:0005198 (structural); GO:0007155 (cell adhesion)
**cldn33b**	Up	claudin 33b	Tight Junction	GO:0070830 (TJ assembly); GO:0005198 (structural); GO:0007155 (cell adhesion)
**tjp3**	Up	tight junction protein 3	Tight Junction	GO:0045216 (junction org.); GO:0150105 (junction localization); GO:0050839 (CAM binding)
**lsr**	Up	lipolysis stimulated lipoprotein receptor	Tight Junction	GO:0060856 (BBB establish.); GO:0061689 (triTJ); GO:1904274 (triTJ assembly)
**LOC134455903**	Up	junctional adhesion molecule A-like	Tight Junction	GO:0090559 (permeability reg.); GO:0007155 (cell adhesion); GO:0005923 (TJ)
**LOC134450241**	Up	claudin-7	Tight Junction	GO:0070830 (TJ assembly); GO:0005198 (structural); GO:0007155 (cell adhesion)
**cxadr**	Up	CXADR Ig-like cell adhesion molecule	Tight Junction	GO:0034109 (homotypic adhesion); GO:0050839 (CAM binding); GO:0005923 (TJ)
**LOC134440051**	Up	cingulin-like protein 1	Tight Junction	GO:0150105 (junction localization); GO:0016459 (myosin complex); GO:0005923 (TJ)
**LOC134452535**	Up	claudin-3-like	Tight Junction	GO:0070830 (TJ assembly); GO:0005198 (structural); GO:0007155 (cell adhesion)
**cldn8.2**	Up	claudin 8.2	Tight Junction	GO:0070830 (TJ assembly); GO:0005198 (structural); GO:0007155 (cell adhesion)
**LOC134453274**	Up	claudin-4-like	Tight Junction	GO:0070830 (TJ assembly); GO:0005198 (structural); GO:0007155 (cell adhesion)
**LOC134453442**	Up	claudin-9-like	Tight Junction	GO:0070830 (TJ assembly); GO:0005198 (structural); GO:0007155 (cell adhesion)
**tjp2a**	Up	tight junction protein 2a (zona occludens 2)	Tight Junction	GO:0045216 (junction org.); GO:0150105 (junction localization); GO:0050839 (CAM binding)
**LOC134445968**	Up	tight junction protein ZO-2-like	Tight Junction	GO:0045216 (junction org.); GO:0150105 (junction localization); GO:0050839 (CAM binding)

**marveld2a**	Up	MARVEL domain containing 2a	Tight Junction	GO:0016324 (apical membrane); GO:0070830 (TJ assembly); GO:0005923 (TJ)
**tjp1b**	Up	tight junction protein 1b	Tight Junction	GO:0045216 (junction org.); GO:0150105 (junction localization); GO:0050839 (CAM binding)
**cldn10e**	Up	claudin 10e	Tight Junction	GO:0070830 (TJ assembly); GO:0005198 (structural); GO:0007155 (cell adhesion)
**cldn1**	Up	claudin 1	Tight Junction	GO:0070830 (TJ assembly); GO:0005198 (structural); GO:0007155 (cell adhesion)
**LOC134454026**	Up	claudin-3-like	Tight Junction	GO:0070830 (TJ assembly); GO:0005198 (structural); GO:0007155 (cell adhesion)
**LOC134452574**	Up	claudin-4-like	Tight Junction	GO:0070830 (TJ assembly); GO:0005198 (structural); GO:0007155 (cell adhesion)
**cldn23.1**	Up	claudin 23.1	Tight Junction	GO:0070830 (TJ assembly); GO:0005198 (structural); GO:0007155 (cell adhesion)
**LOC134461555**	Down	claudin-14-like	Tight Junction	GO:0070830 (TJ assembly); GO:0005198 (structural); GO:0007155 (cell adhesion)
**LOC134452129**	Down	claudin-15-like	Tight Junction	GO:0070830 (TJ assembly); GO:0005198 (structural); GO:0007155 (cell adhesion)
**LOC134442430**	Down	claudin-18-like	Tight Junction	GO:0070830 (TJ assembly); GO:0005198 (structural); GO:0007155 (cell adhesion)
**LOC134443745**	Up	tumor necrosis factor receptor superfamily member 14-like	Cytokine	GO:0007165 (signal transd.); GO:0009897 (ext. membrane); GO:2000406 (T cell migration)
**LOC134456433**	Up	tumor necrosis factor receptor superfamily member 14-like	Cytokine	GO:0009897 (ext. membrane); GO:2000406 (T cell migration); GO:0002720 (cytokine prod.)
**LOC134467646**	Up	butyrophilin subfamily 1 member A1-like	Cytokine	GO:0001817 (cytokine reg.); GO:0009897 (ext. membrane); GO:0050852 (TCR signaling)
**LOC134443912**	Up	tumor necrosis factor receptor superfamily member 14-like	Cytokine	GO:0009897 (ext. membrane); GO:2000406 (T cell migration); GO:0002720 (cytokine prod.)
**wnt7aa**	Up	wingless-type MMTV integration site family, member 7Aa	Cytokine	GO:0046330 (JNK cascade); GO:0045165 (cell fate); GO:0060070 (Wnt signaling)
**bmp16**	Up	bone morphogenetic protein 16	Cytokine	GO:0030509 (BMP signaling); GO:0005125 (cytokine act.); GO:0008083 (GF activity)
**LOC134457579**	Up	protein Wnt-7a	Cytokine	GO:0046330 (JNK cascade); GO:0045165 (cell fate); GO:0060070 (Wnt signaling)
**ifnlr1**	Up	interferon lambda receptor 1	Cytokine	GO:0004896 (cytokine R act.); GO:0019221 (cytokine signaling); GO:0005515 (protein bind.)

**si:dkey-92i15.4**	Up	uncharacterized si:dkey-92i15.4	Cytokine	GO:0050930 (pos. chemotaxis); GO:0042609 (CD4 binding); GO:0030595 (leukocyte chemotaxis)
**LOC134466435**	Up	growth/differentiation factor 8	Cytokine	GO:0008083 (GF activity); GO:0005125 (cytokine act.); GO:0005576 (extracellular)
**LOC134467645**	Up	butyrophilin subfamily 1 member A1-like	Cytokine	GO:0001817 (cytokine reg.); GO:0009897 (ext. membrane); GO:0050852 (TCR signaling)
**LOC134439275**	Up	V-set domain-containing T-cell activation inhibitor 1-like	Cytokine	GO:0001817 (cytokine reg.); GO:0009897 (ext. membrane); GO:0050852 (TCR signaling)
**ifngr1**	Up	interferon gamma receptor 1	Cytokine	GO:0004896 (cytokine R act.); GO:0019221 (cytokine signaling); GO:0005886 (membrane)
**ticam1**	Up	TIR domain containing adaptor molecule 1	Cytokine	GO:0043123 (NF-κB signaling); GO:0035666 (TRIF-TLR signaling); GO:0002735 (DC cytokine prod.)
**bmp2a**	Up	bone morphogenetic protein 2a	Cytokine	GO:0030509 (BMP signaling); GO:0005125 (cytokine act.); GO:0008083 (GF activity)
**LOC134456575**	Up	tumor necrosis factor receptor superfamily member 14-like	Cytokine	GO:0007165 (signal transd.); GO:0009897 (ext. membrane); GO:2000406 (T cell migration)
**il20ra**	Up	interleukin 20 receptor, alpha	Cytokine	GO:0004896 (cytokine R act.); GO:0019221 (cytokine signaling); GO:0005515 (protein bind.)
**wnt2**	Up	wingless-type MMTV integration site family member 2	Cytokine	GO:0045165 (cell fate); GO:0060070 (Wnt signaling); GO:0005125 (cytokine act.)
**prlra**	Up	prolactin receptor a	Cytokine	GO:0009897 (ext. membrane); GO:0019221 (cytokine signaling); GO:0043235 (receptor complex)
**LOC134458237**	Down	interleukin-31 receptor subunit alpha-like	Cytokine	GO:0009897 (ext. membrane); GO:0019221 (cytokine signaling); GO:0043235 (receptor complex)
**bmp15**	Down	bone morphogenetic protein 15	Cytokine	GO:0008083 (GF activity); GO:0005125 (cytokine act.); GO:0005576 (extracellular)
**nfatc2ip**	Down	nuclear factor of activated T cells 2 interacting protein	Cytokine	GO:0001816 (cytokine prod.); GO:0045944 (RNAPII transcr.)
**mstnb**	Down	myostatin b	Cytokine	GO:0008083 (GF activity); GO:0005125 (cytokine act.); GO:0005576 (extracellular)
**LOC134463744**	Down	butyrophilin subfamily 1 member A1-like	Cytokine	GO:0001817 (cytokine reg.); GO:0009897 (ext. membrane); GO:0050852 (TCR signaling)

**crlf1b**	Down	cytokine receptor-like factor 1b	Cytokine	GO:0009897 (ext. membrane); GO:0097058 (CRLF-CLCF1); GO:0019221 (cytokine signaling)
**mif**	Down	macrophage migration inhibitory factor	Cytokine	GO:0050178 (tautomerase act.); GO:0005125 (cytokine act.); GO:0005615 (extracellular)
**csf1b**	Down	colony stimulating factor 1b (macrophage)	Cytokine	GO:0008083 (GF activity); GO:0005125 (cytokine act.); GO:0016020 (membrane)
**macir**	Down	macrophage immunometabolism regulator	Cytokine	GO:1900016 (neg. inflam. cytokine); GO:0010764 (neg. fibroblast migr.)
**LOC134445251**	Up	leukotriene B4 receptor 1-like	Chemokine	GO:0009897 (ext. membrane); GO:0019722 (Ca^{2+} signaling); GO:0019957 (CC chemokine bind.)
**LOC134452030**	Up	monocyte chemotactic protein 1B-like	Chemokine	GO:0006955 (immune resp.); GO:0008009 (chemokine act.); GO:0005576 (extracellular)
**ackr3b**	Up	atypical chemokine receptor 3b	Chemokine	GO:0001525 (angiogenesis); GO:0006935 (chemotaxis); GO:0015026 (coreceptor act.)
**LOC134455011**	Up	C-C motif chemokine 20-like	Chemokine	GO:0006955 (immune resp.); GO:0008009 (chemokine act.); GO:0005576 (extracellular)
**LOC134463391**	Up	C-C chemokine receptor type 6-like	Chemokine	GO:0009897 (ext. membrane); GO:0019722 (Ca^{2+} signaling); GO:0019957 (CC chemokine bind.)
**LOC134458840**	Up	C-C motif chemokine 19-like	Chemokine	GO:0006955 (immune resp.); GO:0008009 (chemokine act.); GO:0005576 (extracellular)
**LOC134441547**	Up	uncharacterized LOC134441547	Chemokine	GO:0006955 (immune resp.); GO:0008009 (chemokine act.); GO:0005576 (extracellular)
**LOC134445482**	Down	C-X-C motif chemokine 11-6-like	Chemokine	GO:0006935 (chemotaxis); GO:0006952 (defense resp.); GO:0006955 (immune resp.)
**LOC134446891**	Down	C-C motif chemokine 5-like	Chemokine	GO:0006955 (immune resp.); GO:0008009 (chemokine act.); GO:0005576 (extracellular)
**LOC134466176**	Down	integrin beta-1-like	Chemokine	GO:0007160 (matrix adhesion); GO:0043236 (laminin bind.); GO:0019960 (CX3C bind.)
**LOC134446892**	Down	C-C motif chemokine 26-like	Chemokine	GO:0006955 (immune resp.); GO:0008009 (chemokine act.); GO:0005576 (extracellular)
**xcr1b.3**	Down	chemokine (C motif) receptor 1b, duplicate 3	Chemokine	GO:0009897 (ext. membrane); GO:0019722 (Ca^{2+} signaling); GO:0019957 (CC chemokine bind.)
**gjb10**	Up	gap junction protein beta 10	Gap Junction	GO:0005922 (connexin complex); GO:0007267 (cell signaling); GO:0005243 (GJ channel act.)

**LOC134464416**	Up	gap junction delta-3 protein-like	Gap Junction	GO:0005922 (connexin complex); GO:0007267 (cell signaling); GO:0086077 (GJ channel act.)
**gjb7**	Up	gap junction protein beta 7	Gap Junction	GO:0005922 (connexin complex); GO:0007267 (cell signaling); GO:0005243 (GJ channel act.)
**LOC134436121**	Up	gap junction beta-4 protein-like	Gap Junction	GO:0005922 (connexin complex); GO:0007267 (cell signaling); GO:0005243 (GJ channel act.)
**gjb9a**	Up	gap junction protein beta 9a	Gap Junction	GO:0005922 (connexin complex); GO:0007267 (cell signaling); GO:0005243 (GJ channel act.)
**LOC134459230**	Up	gap junction gamma-1 protein-like	Gap Junction	GO:0005922 (connexin complex); GO:0007267 (cell signaling); GO:0005243 (GJ channel act.)
**gjb3**	Up	gap junction protein beta 3	Gap Junction	GO:0005922 (connexin complex); GO:0007267 (cell signaling); GO:0005243 (GJ channel act.)
**LOC134469066**	Down	gap junction Cx32.2 protein-like	Gap Junction	GO:0005922 (connexin complex); GO:0007267 (cell signaling); GO:0005243 (GJ channel act.)
**LOC134440480**	Down	gap junction alpha-3 protein-like	Gap Junction	GO:0005922 (connexin complex); GO:0007267 (cell signaling); GO:0005243 (GJ channel act.)
**LOC134469064**	Down	gap junction Cx32.2 protein-like	Gap Junction	GO:0005922 (connexin complex); GO:0007267 (cell signaling); GO:0005243 (GJ channel act.)

Supplementary Figures

(A)

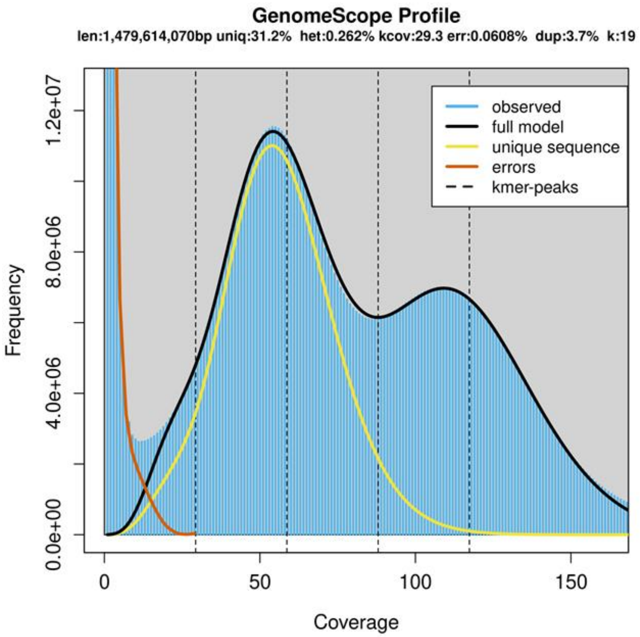

(B)

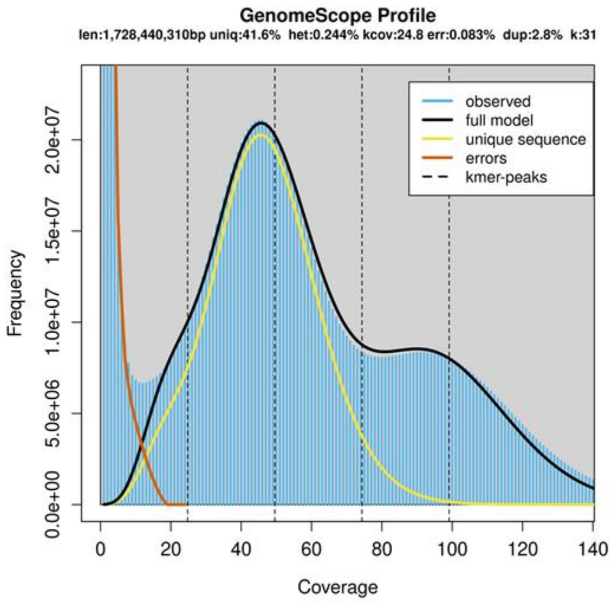

Property	***k-mer* 19**	***k-mer* 31**
Heterozygosity	0.273938%	0.253823%
Genome Haploid Length	1,479,614,070 bp	1,728,440,310 bp
Genome Repeat Length	1,017,989,440 bp	1,009,883,392 bp
Genome Unique Length	461,624,630 bp	718,556,918 bp
Model Fit	99.1971%	97.3636%
Read Error Rate	0.0608218%	0.0829669

Figure S1. K-mer frequency distribution of *E. encrasicolus* using Illumina short reads. The K-mers were counted using Jellyfish with the parameter "-C -m 51 -s 10000000000 -t 96" and result file (".*histo*") imported in GenomeScope (tohttp://genomescope.org/) to plot the frequency. Genome size can be estimated as (total K-mer number) / (the volume peak). **(A)** K-mer (19-mer) based estimation of genome characters of *E. encrasicolus*. **(B)** K-mer (31-mer) based estimation of genome characters of *E. encrasicolus*.

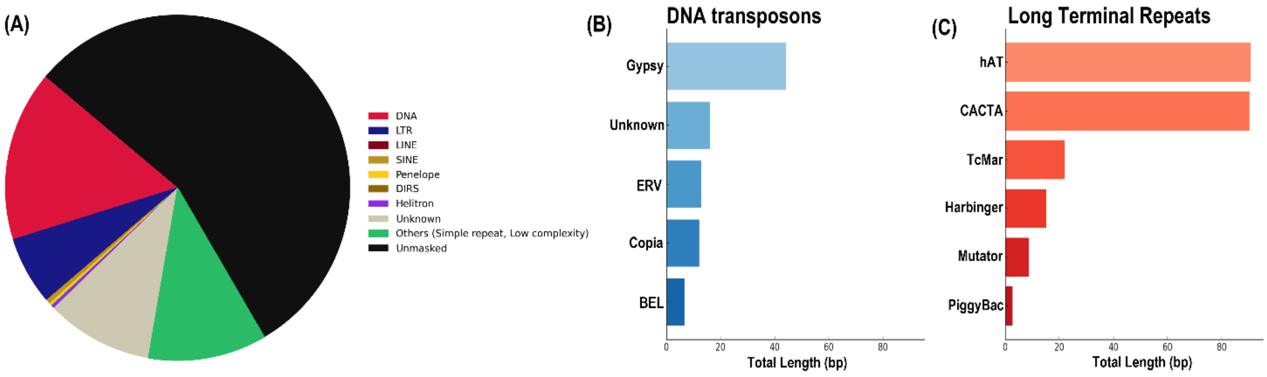

Figure S2. Repeat content of the *E. encrasicolus* genome. **(A)** Proportion of repeats and masked regions within the genome, **(B)** Length distribution of DNA transposons, **(C)** Length distribution of long terminal repeats.
